# Dissociative seizures mimicking epileptic seizures: diagnostic challenges in a case with atypical eye movements

**DOI:** 10.1186/s42494-025-00210-w

**Published:** 2025-03-04

**Authors:** Shimin Bao, Caleb Onyenaturuchi Egbuta, Jinmei Li

**Affiliations:** 1 Department of Neurology, West China Hospital, Sichuan University, Chengdu, 610041 China; 2 Department of Neurology, Hospital of Chengdu Office of People’s Government of Tibetan Autonomous Region (Hospital.C.T.), Chengdu, 610041 China

**Keywords:** Dissociative seizures, Epileptic seizures, Psychogenic nonepileptic seizures, Video-electroencephalogram, Depression, Oculogyric crisis

## Abstract

**Background:**

Dissociative seizures (DS), also known as psychogenic non-epileptic seizures (PNES), often mimic epileptic seizures (ES), leading to misdiagnosis, unnecessary anti-seizure medications (ASMs)/ suboptimal use of ASMs, and delays in appropriate care in approximately one-third of patients. Rare presentations, such as episodes resembling oculogyric crisis (OGC), further complicate differentiation. This report highlights the diagnostic challenges of DS with atypical features and emphasises the role of video-electroencephalogram (VEEG) in early differentiation.

**Case presentation:**

We present a 16-year-old male with recurrent episodes of upward eye deviation, non-synchronised limb twitching, and bizarre behaviours, initially misdiagnosed as epilepsy and autoimmune encephalitis. Comprehensive investigations, including normal neuroimaging, absence of epileptiform activity on VEEG, and psychological evaluation revealing moderate depression, supported a diagnosis of DS. The patient showed significant improvement with sertraline and cognitive behavioural therapy.

**Conclusions:**

This case underscores the diagnostic challenges posed by atypical DS presentations and highlights the value of/need for VEEG and psychiatric evaluation in differentiation. Early identification of DS can prevent mismanagement and optimize outcomes.

## Background

Dissociative seizures (DS), also known as psychogenic non-epileptic seizures (PNES), are episodes of altered motor, sensory, or behavioural function that mimic epileptic seizures (ES) but lack an epileptiform brain activity basis [1, 2]. While they can present with a wide range of symptoms, they are often characterised by prolonged duration, asynchronous movements, forced eye closure, and emotional expression during or after the episode [3].Misdiagnosis of DS as ES is a common challenge, leading to unnecessary exposure to anti-seizure medications (ASMs), invasive investigations, and psychological distress. Accurate and timely diagnosis is crucial to initiate appropriate treatment, which typically involves psychological therapies such as cognitive behavioural therapy (CBT) and, in some cases, psychopharmacological interventions [1, 4].

The exact etiology of dissociative seizures remains unclear, but psychological factors, such as stress, trauma, and emotional distress, are often implicated [5]. Understanding the underlying mechanisms and triggers of these seizures is essential for developing effective treatment strategies and improving patient outcomes. The diagnosis of DS relies on a combination of clinical evaluation, detailed history-taking, and confirmatory video-electroencephalogram (VEEG) monitoring. VEEG is considered as the gold standard for differentiating DS from ES, as it allows for simultaneous recording of brain electrical activity and clinical manifestations [6].

Oculogyric crisis (OGC) is a neurological condition characterised by involuntary, sustained upward deviation of the eyes. It can be associated with various neurological disorders, including Parkinson’s disease, drug-induced dystonia, and encephalitis [7]. However, it is important to note that OGC can also be a manifestation of DS. In such cases, the eye deviation may be accompanied by other non-epileptic features, such as emotional expression, forced eye closure, or bizarre motor behaviours [5, 8].

## Case presentation

We present the case of a 16-year-old right-handed male with a three-month history of episodic upward eye deviation, non-synchronised limb twitching, and bizarre behaviours. The episodes were characterised by a backward thrust of the head, painful facial expressions, photophobia, dyspnoea, and frequent screaming, without loss of consciousness. The patient was initially diagnosed with ES and autoimmune encephalitis at a different hospital, based on the detection of serum anti-Recoverin and anti-GQ1b antibodies. Treatment with ASMs (e.g., levetiracetam, valproic acid), intravenous immunoglobulin (IVIG), and rituximab, proved ineffective.

Upon referral to our hospital, neurological examination, brain magnetic resonance imaging (MRI), and computerized tomography (CT) imaging revealed no abnormalities. VEEG monitoring recorded the episodes but showed no epileptiform discharges, with only movement artefacts observed. Psychological assessment identified the self-rating anxiety scale (SAS) score of 46 (indicating no anxiety tendencies) and the self-rating depression scale (SDS) score of 63 (consistent with moderate depression). A comprehensive autoimmune panel, including serum and cerebrospinal fluid (CSF) testing for neural autoantibodies associated with autoimmune encephalitis, yielded negative results. These findings, combined with the patient’s clinical presentation, led to a diagnosis of DS. The patient was prescribed sertraline (50 mg daily) and referred to the mental health department for CBT. CBT was conducted by a psychiatrist for two months, and focused on cognitive restructuring, helping the patient view his symptoms more rationally and reduce unnecessary fear and anxiety. Behavioural training included systematic desensitisation, relaxation techniques, and emotion regulation strategies to help the patient gradually regain function. At telephone follow-up, the patient reported adherence to treatment, with significant symptomatic improvement and only one fleeting episode of mild convulsion. He had resumed daily activities and school with a positive outlook.

## Discussion

DS represent a heterogenous group of episodic disorders characterised by altered subjective experiences, involuntary motor phenomena, and a transient loss of self-control. Unlike ES, which result from abnormal neuronal discharge, DS arise from complex neuropsychiatric dysfunctions. This distinction underpins their classification as dissociative disorders in the International Classification of Diseases (ICD) [9, 10].. Despite the absence of epileptiform activity, DS can exhibit features that closely mimic ES, such as tonic–clonic limb movements and eye-motor crises. These overlapping clinical manifestations frequently complicate the diagnostic process, leading to delayed or inappropriate management.

This case illustrates the diagnostic challenge posed by DS when atypical features, such as OGC-like episodes, are present. OGC, characterised by tonic upward eye deviation, is typically associated with dystonic disorders, neurometabolic or neurodegenerative conditions, or adverse reactions to medications such as antipsychotics and ASMs [7]. However, DS episodes may manifest with pseudo-OGC features, further confounding the diagnostic process [11]. Misdiagnosis of DS as ES or other neurological disorders is common and can result in inappropriate treatments, such as unnecessary ASMs or immunomodulatory therapies, as observed in this patient [6]. The duration of DS episodes often exceeds that of ES, and psychiatric comorbidities such as post-traumatic stress disorder (PTSD), borderline personality disorder, and mood disorders frequently co-occur, reflecting the complex neuropsychiatric underpinnings of DS [11–13].

This case highlights the importance of adopting a structured diagnostic approach integrating VEEG, psychiatric evaluation, and detailed clinical observation to improve diagnostic accuracy and guide appropriate management.

### Differential diagnosis

The patient’s initial presentation with upward eye deviation and limb twitching led to diagnostic uncertainty, as these features may mimic OGC, a phenomenon associated with dystonic disorders or antipsychotic medications. However, the absence of tonic eye deviation on VEEG, the presence of forced eye closure, and bizarre behaviours were more consistent with DS. Forced eye closure is a hallmark of DS and is rarely observed in ES or OGC [7, 14–16]. Comprehensive laboratory and CSF analyses, which ruled out autoimmune encephalitis, further underscored the need to shift the diagnostic focus from an organic etiology to a functional disorder.

### Psychiatric considerations

Psychological evaluation revealed moderate depression, a prevalent comorbidity in DS, supporting the diagnosis. Depression and other mood disorders are frequently implicated in the pathophysiology of DS and may serve as both risk factors and perpetuating factors. Addressing these underlying psychological disturbances is critical for effective management. CBT and pharmacological interventions, such as sertraline in this case, have demonstrated efficacy in improving outcomes.

### The role of VEEG

VEEG remains the gold standard for distinguishing DS from ES. In this patient, the lack of epileptiform activity during ictal and interictal periods, coupled with artefacts correlating to movement patterns, was instrumental in confirming the diagnosis. Early use of VEEG can significantly reduce the time to diagnosis, prevent unnecessary treatments, and minimise the psychological and physical burden on patients.

### Clinical implications

This case underscores the need for improved clinical awareness of DS, particularly its atypical presentations. Incorporating a multidisciplinary approach, including neurologists, psychiatrists, and psychologists, is essential for accurate diagnosis and comprehensive care. Additionally, clinicians should be vigilant about comorbid psychiatric conditions, as their treatment can substantially influence DS outcomes.

A comparative table highlighting the key differences between DS, ES, and OGC can aid in the differential diagnosis (Table 1). As seen in Table 1, the patient's clinical features, such as the prolonged duration of episodes, bizarre movements, preserved consciousness, and emotional expression, align more closely with the characteristics of DS rather than ES or OGC.

**Table 1:** Comparative Semiology of Dissociative Seizures (DS), Epileptic Seizures (ES), and Oculogyric Crisis (OGC). (Adapted from Mellers et al. [17])

Feature	Dissociative Seizures (DS)	Epileptic Seizures (ES)	Oculogyric Crisis (OGC)
Etiology	Psychological (e.g., stress, trauma, conversion disorder)	Neurological (e.g., abnormal neuronal discharges)	Neurological or pharmacological (e.g., dopamine blockade)
Eye movements	Forced eye closure, inconsistent or fluctuating eye movements	Sustained eye deviation (e.g., tonic deviation during seizures)	Persistent upward or lateral gaze deviation
EEG findings	Normal during ictal and interictal periods	Ictal or interictal epileptiform discharges	Normal EEG, no epileptiform discharges
Movement patterns	Bizarre, asynchronous, or exaggerated movements	Stereotypical, rhythmic, and synchronised motor activity	Dystonic posturing without rhythmic movements
Consciousness	Preserved or variably altered, with memory recall often intact	Altered or lost consciousness, typically with postictal confusion	Fully conscious during episodes
Emotional expression	Prominent (e.g., crying, grimacing, fear, or anger)	Minimal to absent emotional involvement during ictal events	No emotional overlay
Duration	Longer duration, with gradual onset and termination	Brief, typically less than 2 min	Episodes lasting minutes to hours
Response to treatment	Poor response to ASMs, responsive to psychological therapy (e.g., CBT)	Responsive to appropriate ASMs	Responsive to anticholinergics or dopamine agonists
Trigger factors	Psychological stress, trauma, or emotional events	Sleep deprivation, flashing lights, or metabolic derangements	Medications (e.g., antipsychotics) or metabolic causes
Post-episode state	Rapid recovery, no postictal confusion	Postictal drowsiness or confusion common	Persistent dystonia may linger post-episode
Comorbidities	High prevalence of mood disorders (e.g., depression, anxiety)	Variable, depending on epilepsy type	Parkinsonism, neuroleptic use, or encephalopathy

*Abbreviations: EEG* Electroencephalogram, *ASMs* Anti-seizure medications, *CBT* Cognitive behavioural therapy

While this case highlights the importance of early diagnosis and appropriate management of DS, it is important to acknowledge the limitations of this study. The small sample size and short follow-up period limit the generalizability of the findings. Further research is needed to elucidate the underlying pathophysiology of DS, develop standardised diagnostic criteria and biomarkers, and evaluate the long-term efficacy of various treatment approaches. Additionally, enhanced education and awareness campaigns can help reduce stigma and improve recognition of DS in clinical practice.

## Conclusions

This case report underscores the diagnostic challenges posed by atypical presentations of DS, especially when they mimic neurological conditions such as OGC. It also emphasizes the importance of a comprehensive diagnostic approach, including a detailed clinical history, neurological examination, neuroimaging studies, and most importantly, VEEG monitoring. Recognizing the diverse clinical manifestations of DS, including atypical presentations, is crucial for accurate diagnosis and appropriate management.

## Data Availability

All data generated during this study are included in this article. Further enquiries can be directed to the corresponding author.

## Abbreviations

ASMs: Anti-seizure medications
CBT: Cognitive behavioural therapy
DS: Dissociative seizures
ES: Epileptic seizures
OGC: Oculogyric crisis
PNES: Psychogenic non-epileptic seizures
VEEG: Video-electroencephalogram
IVIG: Intravenous immunoglobulin
MRI: Magnetic resonance imaging
CT: Computerized tomography
CSF: Cerebrospinal fluid
PTSD: Posttraumatic stress disorder
ICD: International Classification of Diseases
